# Correlation of the Neutrophil-to-lymphocyte and Platelet-to-lymphocyte Ratios with Postoperative Complications and Survival in Surgery for Bone Metastasis of the Appendicular Skeleton

**DOI:** 10.1055/s-0045-1804497

**Published:** 2025-04-11

**Authors:** Matheus Silva Teixeira, Ana Valeria Rigolino Teixeira, Glauco Jose Pauka Mello, Fernando Issamu Tabushi, Claudio Luciano Franck, Carmen Austrália Paredes Marcondes Ribas

**Affiliations:** 1 Serviço de Oncologia Ortopédica, Hospital Erasto Gaertner, Curitiba, PR, Brasil; 2 Faculdade Evangélica Mackenzie do Paraná, Curitiba, PR, Brasil

**Keywords:** blood platelets, lymphocytes, neoplasm metastasis, neutrophils, operative surgical procedures, postoperative complications

## Abstract

**Objective** To analyze, in cases of long-bone metastases, the incidence of postoperative complications and survival of up to 1 year, correlating them with the neutrophil-to-lymphocyte ratio (NLR) and platelet-to-lymphocyte ratio (PLR).

**Methods** Review of 160 medical records of patients who underwent surgery for bone metastases in the appendicular skeleton. We determined epidemiological characteristics and NLR and PLR values, which were correlated with survival and complications.

**Results** Women represented 64.5% of the sample, and 62.6% presented primary breast tumor. The proximal femur was the most affected bone. The median survival was of 13.2 months, and the 1-year survival rate, of 34.7%. Tumor resection with endoprosthesis was the most common surgery. The postoperative complication rate was of 10%, and the mean time until occurrence was of 27.9 (range: 0–140) days. We observed a significant association between neutrophil levels and postoperative complications ( *p* = 0.04): for every increase of 100 neutrophils, the risk of postoperative complications increased by 1%. The mean NLR and PLR values were of 5.3 (range: 0.2–30.7) and 199.7 (range: 32.1–676.7) respectively. Patients with NLR ≥ 2 ( *p* < 0,001) showed a decrease in survival from 92,3 to 62,5% in the third month, and from 61,5 to 31,3% in the first year. Those with PLR ≥ 209 ( *p* < 0.001) showed a decrease in survival from 69 to 59.3% in the third month, and from 40.2 to 25.9% in the first year.

**Conclusion** There was no positive association regarding the NLR and PLR and postoperative complications. However, we noted a strong correlation involving elevated NLR and PLR levels and reduced life expectancy starting from the third postoperative month.

## Introduction

Cancer is a public health problem, with approximately 626 thousand new cases in Brazil in 2020. 1 Bone metastases (BMs) are the most common malignant neoplasm of the skeleton, ranking third in the spread of adenocarcinomas after the lung and the liver. 2 3 Bone metastasis occurs mainly in the axial skeleton (80%), pelvis, and femur, especially affecting women after the fourth decade of life. 4 5 Most BMs originate from breast, prostate, or lung cancers. 4 5

Bone metastasis is the main cause of morbidity in patients with advanced cancer, 6 and its control is essential to improve the patient's quality of life, pain, and independence. 2 Treatment is multidisciplinary, including clinical or surgical measures.

Surgery aims to stabilize imminent or pathological fractures, promote pain reduction, improve limb function, provide early ambulation, and avoid complications of prolonged decubitus. 2 The surgical decision must consider metastasis location and extension, the response to adjuvant therapies, the clinical condition, and the life expectancy. 6 The complication rate in BM treatment correlates with advanced age, clinical comorbidities, immunodeficiency, malnutrition, prolonged hospitalization, and local irradiation. 7

Inflammation is a hallmark of cancer. In some tumors, it precedes malignancy; in others, the tumor induces an inflammatory response, favoring its growth. 8 Inflammatory biomarkers, such as the neutrophil-to-lymphocyte ratio (NLR) and platelet-to-lymphocyte ratio (PLR), are prognostic in several cancer types. 9 Increased NLR correlate with lower survival in multiple myeloma and gastric, colorectal, lung, breast, and endometrial neoplasms. 10 Therefore, the NLR and PLR may be used to predict survival and postoperative complications in patients with BM. 2

The present study aimed to identify the epidemiological profile of patients with metastasis in long bones, the complication rate, the postoperative survival rate, and the correlation of the NLR and PLR with complications and survival up to 1 year postoperatively.

## Materials and Methods

For the current retrospective, longitudinal, analytical, and descriptive observational study, we selected patients from the surgical registry book of the Orthopedic Oncology Service of Hospital Erasto Gaertner and extracted data by reviewing the Tasy (Philips Healthcare, Best, Netherlands) electronic medical records per the Brazilian General Personal Data Protection Law (Lei Geral de Proteção de Dados Pessoais, LGPD, in Portuguese; law no. 13,709/2018). The data was collected from patients who underwent surgery from January 1, 2010, to December 31, 2019.

The epidemiological profile included age, gender, primary tumor site, BM location, surgical procedure, presence of other metastases, comorbidity, and previous radiotherapy (RT). We determined the NLR and PLR and their correlation with survival and postoperative complications.

The inclusion criteria were patients who underwent surgery for appendicular skeleton BM with confirmed diagnosis of metastatic malignant bone neoplasm by histological or immunohistochemical examination. The exclusion criteria were incomplete medical records, patients submitted to revision surgery in another institution, and absence of preoperative blood count.

We obtained the NLR and PLR from the preoperative blood count performed in the hospital laboratory up to 72 hours before surgery. We considered data from the first surgical procedure alone for patients undergoing multiple orthopedic surgeries due to BM.

### Statistical Analysis

We organized the data in an Excel (Microsoft Corp., Redmond, WA, United States) spreadsheet and analyzed it using the Stata/SE software (StataCorp L LC, College Station, TX, United States), version 14.1. The quantitative variables were expressed as mean, standard deviation, median, minimum, maximum, and interquartile range (IQR) values. The categorical variables were expressed as absolute frequencies and percentages. Adjusted logistic regression models were used to analyze the factors associated with postoperative complications. The Wald test was applied to assess the significance of each variable. The estimated measure of association was the odds ratio (OR) with 95% confidence intervals (95%CIs).

The Cutoff Finder software, 11 which identifies the cutoff values generating significant differences in the log-rank tests, determined the cutoff points for NLR and PLR. This process ensured that the selected cutoff points corresponded to the NLR and PLR levels most related to the selected clinical outcomes. Statistical significance was set at *p* < 0.05.

## Results

### Epidemiological Profile

We evaluated a total of 160 medical records from 2010 to 2019. Of these, we excluded 16 due to lack of data or loss to follow-up, 2 due to lack of metastasis confirmation in the anatomopathological examination, and 1 due to surgery in another service. We included a total of 154 surgeries, and 12 patients presented multiple metastases. For data analysis, we considered only the first surgery, totaling 141 procedures.

The mean age of the patients was of 61.5 (range: 25–89) years, and the mean survival time was of 13.2 (range: 0–99.6) months. The mean laboratory parameters were within normal limits per blood count reference values. 12 The NLR was of 5.3 (range: 0.2–30.7), and the PLR, of 199.7 (range: 32.1–676.7; Table 1 ).

**Table 1:** Quantitative epidemiological variables

Variable	n	Mean	Standard deviation	Median	Minimum	Maximum	Interquartile range
Age (years)	141	61.5	12	62	25	89	54–69
Survival (months)	141	13.2	18.8	5	0	99.6	1.9–16.9
Neutrophils	141	6,451	3,094	5,762	137	19,272	4,276–8,398
Lymphocytes	141	1,542	779	1,315	301	4,518	999–1,958
Leukocytes	141	8,657	3,486	8,100	980	21,900	6,340–10,800
Platelets	141	252,579	104,642	241,000	63,000	596,000	174,000–308,000
Neutrophil-to-lymphocyte ratio	141	5.3	4.5	4.2	0.2	30.7	2.7–6.3
Platelet-to-lymphocyte ratio	141	199.7	118.8	171.6	32.1	676.7	114.9–248

Regarding gender, 64.5% (n = 91) of the subjects were women. The femur was the most affected bone (n = 112), followed by the humerus (n = 21). As for the surgical procedure, 67% of the patients underwent tumor resection with hip endoprosthesis replacement. Most subjects (80.1%) presented multiple BMs, and 51.1% presented visceral metastases. Table 2 summarizes the remaining data.

**Table 2:** Categorical epidemiological variables

Variable	Classification	n	%
Gender	Female	91	64.50%
	Male	50	35.50%
Anatomy	Diaphyseal femur	3	2.10%
	Distal femur	10	7.10%
	Proximal femur	99	70.20%
	Radius	2	1.40%
	Diaphyseal tibia	1	0.70%
	Proximal tibia	5	3.50%
	Diaphyseal humerus	8	5.70%
	Distal humerus	2	1.40%
	Proximal humerus	11	7.80%
Surgery	Hip endoprosthesis	95	67.40%
	Knee endoprosthesis	15	10.60%
	Shoulder endoprosthesis	10	7.10%
	Fracture fixation	6	4.30%
	Arthroplasty resection	4	2.80%
	Diaphyseal femoral endoprosthesis	3	2.10%
	Elbow endoprosthesis	2	1.40%
	Amputation	1	0.70%
	Disarticulation	1	0.70%
	Total femoral endoprosthesis	1	0.70%
	Diaphyseal humeral endoprosthesis	1	0.70%
	Proximal radial resection	1	0.70%
	Intralesional resection	1	0.70%
Presence of other visceral metastasis	No	72	51.10%
	Yes	69	48.90%
Presence of other bone metastasis	No	28	19.90%
	Yes	113	80.10%
Diabetes	No	110	78.00%
	Yes	31	22.00%
Previous radiotherapy	No	136	96.50%
	Yes	5	3.50%

Table 3 shows the distribution and categorization of the primary tumor. In female patients, the most common primary site was the breast, with 62.6% (n = 57); in male subjects, the most common primary tumor was the prostate, with 38% (n = 19). In both genders, the second most common neoplasm was lung cancer.

**Table 3:** Gender and primary diagnosis

Primary diagnosis	Gender				Total	
	Female		Male			
	n	%	n	%	n	%
Breast cancer	57	62.60%			57	40.40%
Prostate cancer			19	38.00%	19	13.50%
Lung and adnexa cancer	9	9.8	9	18.00%	18	12.70%
Kidney cancer	5	5.50%	9	18.00%	14	9.90%
Unknown primary diagnosis	7	7.70%	1	2.00%	8	5.70%
Colon cancer	1	1.10%	2	4.00%	3	2.10%
Endometrium cancer	3	3.30%			3	2.10%
Gastric cancer	2	2.20%	1	2.00%	3	2.10%
Bladder cancer	2	2.20%			2	1.40%
Esophagus cancer			2	4.00%	2	1.40%
Skin cancer			2	4.00%	2	1.40%
Pharynx cancer			1	2.00%	1	0.70%
Gastrointestinal cancer	1	1.10%			1	0.70%
Intracranial hemangiopericytoma	1	1.10%			1	0.70%
Liver cancer			1	2.00%	1	0.70%
Larynx cancer			1	2.00%	1	0.70%
Melanoma	1	1.10%			1	0.70%
Oropharynx cancer			1	2.00%	1	0.70%
Pancreas cancer	1	1.10%			1	0.70%
Thyroid cancer			1	2.00%	1	0.70%
Uterus and adnexa cancer	1	1.10%			1	0.70%
Total	91	100%	50	100%	141	100%

### Postoperative Complications

Table 4 shows that the mean time until the development of postoperative complications was of 27.9 (range: 0–140) days. The complication rate was of 31.2% (n = 44). Table 5 shows that the most common complications were pneumonia (n = 10) and surgical wound infection (n = 5). A total of 4 patients presented deep infection, including 2 with prosthesis dislocation. All underwent surgical cleaning and debridement, and two were submitted to a new surgery for implant replacement.

**Table 4:** Time until postoperative complication

Variable	n	Mean	Standard deviation	Median	Minimum	Maximum	Interquartile range
Time until postoperative complication (days)	44	27.9	31.7	15	0	140	3–42

**Table 5:** Incidence of postoperative complications

Variable	Classification	n	%
Postoperative complications	No	97	68.8%
	Yes	44	31.2%
Type of postoperative complication (n = 44)	Pneumonia	10	22.7%
	Surgical wound infection	5	11.3%
	Gastrointestinal tract bleeding	4	9.1%
	Prosthesis infection	4	9.1%
	Prosthesis dislocation	4	9.1%
	Acute arterial occlusion in lower limb	2	4.5%
	Surgical wound bleeding	2	4.5%
	Pulmonary thromboembolism	2	4.5%
	Hemodynamic shock	2	4.5%
	Local recurrence	2	4.5%
	Hypercalcemia	2	4.5%
	Dehiscence with material exposure	1	2.3%
	Urinary tract infection	1	2.3%
	Systemic inflammatory response syndrome	1	2.3%
	Deep vein thrombosis	1	2.3%

### Assessment of the Association between Factors and Postoperative Complications

For each variable, we tested the null hypothesis of association with the probability of postoperative complications versus the alternative hypothesis of the existence of an association. Table 6 presents the demographic and clinical factors and the postoperative complications.

**Table 6:** Demographic and clinical factors and postoperative complications

Variable	Classification	Total	Postoperative complications		p*	OR (95%CI)
			No	Yes		
Age (years)	Mean ± SD	141	60.7 ± 12.5	63.3 ± 10.9	0.238	1.02 (0.99–1.05)
Gender	Male	50	39 (78.0%)	11 (22.0%)		
	Female	91	58 (63.7%)	33 (36.3%)	0.083	2.02 (0.91–4.46)
Presence of other visceral metastases	No	72	46 (63.9%)	26 (36.1%)		
	Yes	69	51 (73.9%)	18 (26.1%)	0.201	1.60 (0.78–3.29)
Presence of other bone metastases	No	28	19 (67.9%)	9 (32.1%)		
	Yes	113	78 (69%)	35 (31.0%)	0.905	1.06 (0.44–2.57)
Diabetes	No	110	76 (69.1%)	34 (30.9%)		
	Yes	31	21 (67.7%)	10 (32.3%)	0.886	0.94 (0.40–2.21)
Previous radiotherapy	No	136	94 (69.1%)	42 (30.9%)		
	Yes	5	3 (60.0%)	2 (40.0%)	0.668	0.67 (0.11–4.16)
Neutrophils (every 100)	Median (IQR)	141	5,550 (3,760–7,900)	6,941 (4,933–8,689)	0.040	1.01 (1.001–1.02)
Lymphocytes (every 100)	Median (IQR)	141	1,280 (974–1,940)	1,361 (1,058–2,005)	0.925	1.00 (0.96–1.05)
Leukocytes (every 100)	Mean ± SD	141	8,280 ± 3,246	9,487 ± 3,876	0.062	1.01 (1.00–1.02)
Platelets (every 10,000)	Median (IQR)	141	237,000 (181,000–304,000)	243,500 (154,000–310,000)	0.797	1.00 (0.96–1.03)
Hemoglobin	Mean ± SD	141	11.9 ± 2.0	11.3 ± 1.8	0.145	0.87 (0.72–1.05)
**Neutrophil-to-lymphocyte ratio**	**Median (IQR)**	**141**	**4.1 (2.5–5.8)**	**4.5 (3.2–6.9)**	**0.534**	**1.03 (0.95–1.11)**
**Platelet-to-lymphocyte ratio**	**Median (IQR)**	**141**	**167 (116–269)**	**177 (105–242)**	**0.534**	**0.999 (0.996–1.002)**

**Abbreviations:** 95%CI, 95% confidence interval; OR, odds ratio; IQR, interquartile range; SD, standard deviation.

**Note:** *Logistic regression model and Wald test; *p* < 0.05

There was no correlation of hemoglobin levels, diabetes, and previous RT with complications. Neither did the presence of other metastases, visceral or bone, correlate with complications. For neutrophils alone, there was a significant association with the probability of complications ( *p* = 0.040): for every 100 additional neutrophils, the chance of complications increased by 1%. The NLR and PLR were not risk factors for complications.

### Death

#### Descriptive Analysis of Survival Time

**Fig. 1:**
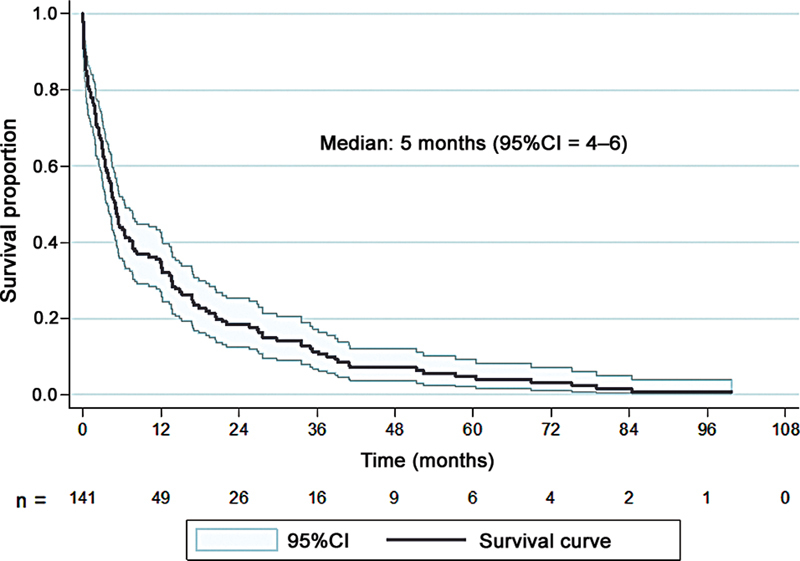
Kaplan-Meier curve with patient survival time (95% confidence interval [95%CI]). Definition of neutrophil-to-lymphocyte ratio (NLR) and platelet-to-lymphocyte ratio (PLR) cutoff points associated with survival time.

Fig. 1 presents the Kaplan-Meier curve for the survival time and the median survival time, which was of 5 months, with a 1-year survival rate of 34.7% (n = 49).

**Fig. 2:**
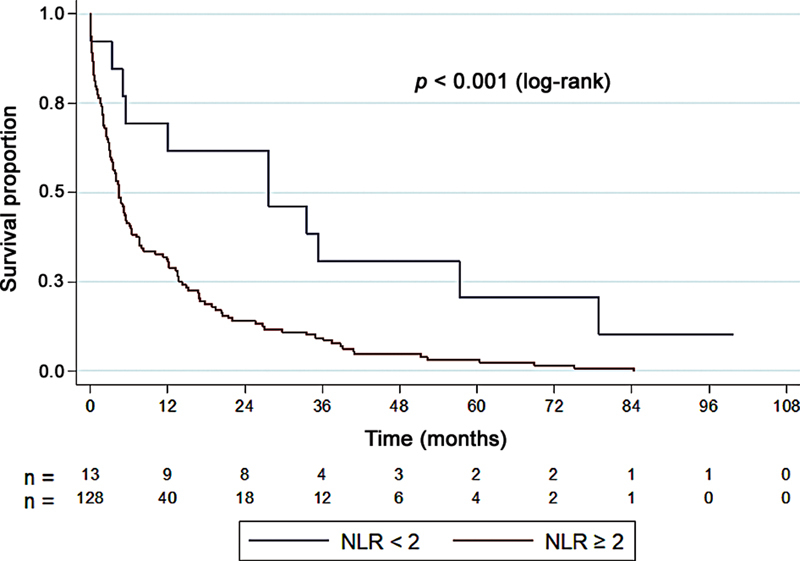
Survival curve considering an NLR cutoff point of 2.

The most significant cutoff values were NLR = 2 and PLR = 209. Table 7 and Fig. 2 show the survival rates and Kaplan-Meier curves considering the cutoff point of NLR = 2. We tested the null hypothesis that the survival curves were the same for NLR < 2 and NLR ≥ 2 against the alternative hypothesis that they differed. From the third postoperative month onwards, the survival of patients with NLR ≥ 2 ( *p* < 0.001) decreased from 92.3 to 62.5% and, at 12 months, from 61.5 to 31.3% ( Table 8 ).

**Table 7:** Survival rate considering the neutrophil-to-lymphocyte ratio cutoff point of 2

Variable	Classification	Total	Death				*p* *
			No		Yes		
			n	%	n	%	
Neutrophil-to-lymphocyte ratio	< 2	13	2	15.4%	11	84.6%	< 0.001
	≥ 2	128	0	0%	128	100%	

**Note:** *Log-rank test; *p* < 0.05.

**Table 8:** Survival percentages estimated by the Kaplan-Meier curve for NLR <2 and ≥ 2

Time	Survival (%)	
	NLR < 2	NLR ≥ 2
0 = surgery	100%	100%
1 month	92.3%	92.2%
3 months	92.3%	62.5%
6 months	69.2%	41.4%
12 months	61.5%	31.3%

Abbreviation: NLR, neutrophil-to-lymphocyte ratio.

**Fig. 3:**
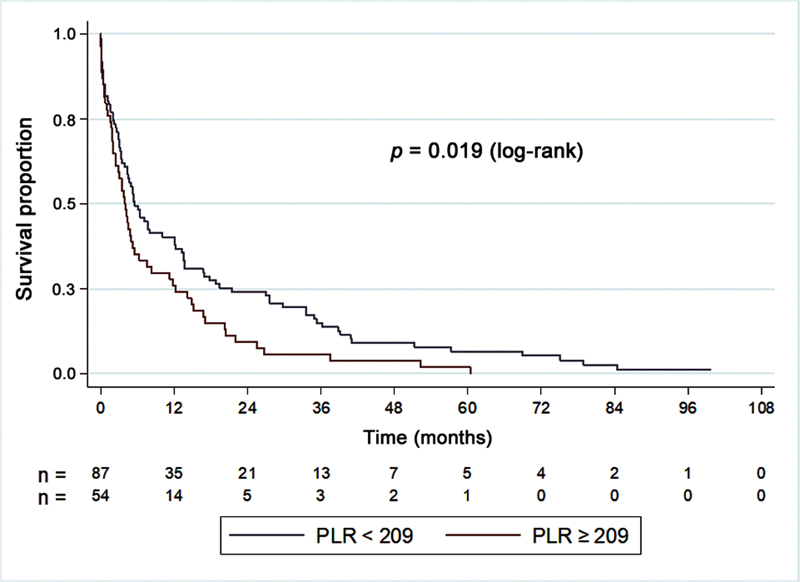
Survival curve considering a PLR cutoff point of 209.

For the PLR, we tested the null hypothesis that the survival curves were the same for cases with PLR < 209 and PLR ≥ 209 versus the alternative hypothesis that they were different. Similarly to the NLR, patients with PLR ≥ 209 ( *p* < 0.001) presented a lower survival rate, decreasing from 69 to 59.3% in the third month, and from 40.2 to 25.9% 1 year postoperatively ( Table 9 ). Table 10 and Fig. 3 show the results and Kaplan-Meier survival curves considering the cutoff point of PLR = 209.

**Table 9:** Survival rate considering the platelet-to-lymphocyte ratio cutoff point of 209

Variable	Classification	Total	Death				*p* *
			No		Yes		
			n	%	n	%	
Platelet-to-lymphocyte ratio	< 209	87	2	2.3%	85	97.7%	0.019
	≥ 209	54	0	0%	54	100%	

**Note:** *Log-rank test; *p* < 0.05.

**Table 10:** Survival percentages estimated by the Kaplan-Meier curve for PLR < 209 and ≥ 209

Time	Survival (%)	
	PLR < 209	PLR ≥ 209
0 = surgery	100%	100%
1 month	81.6%	79.6%
3 months	69.0%	59.3%
6 months	49.4%	35.2%
12 months	40.2%	25.9%

Abbreviation: PLR, platelet-to-lymphocyte ratio.

## Discussion

More than 18 million cases of cancer are registered annually worldwide, and approximately 50% of these subjects will develop advanced metastatic disease. 13 14 Advances in oncological treatment increased patient survival and, at the same time, the number of BM cases. According to estimates, the prevalence of BM in the United States will reach 600 thousand by 2030. 4

The present study included 141 patients undergoing surgical treatment for BM, with a mean age of 61.5 (range: 25 to 89) years and a predominance of women (64.5%; n = 91). Meohas et al. 15 and Li et al. 16 observed a higher incidence in women over 40 years of age.

Hernández et al. 17 analyzed 382,733 patients with primary solid tumors. The incidence of BM was of 6.9% (n = 26,250), and the mean age of the patients was of 64 years. The most prevalent tumors and those with the highest susceptibility to BM were breast, prostate, and lung cancer. 17 In another study 18 with 85,296 patients with BM, the prevalence was of 64.2% for breast tumors, of 59.89% for prostate tumors, of 52.85% for nasopharynx cancers, of 35.82% for lung cancers, and of 35.10% for kidney cancers. Consistent with the literature, in the present study, 66% of BMs originated from breast, prostate, and lung tumors.

More than 80% of BM cases are in the axial skeleton, especially in the vertebrae, ribs, and hips. 19 In the appendicular skeleton, BM frequently involves the proximal regions of the limbs. Lesions below the knee and elbow are rare and associated with lung, kidney, and thyroid neoplasms. 20 In a study 21 with 171 patients, 58 presented appendicular lesions, including 73.8% in the proximal femur, 58.3% in the proximal humerus, and only 4 cases in the tibia. In the current analysis, the most affected bone was the femur, with 112 cases, followed by the humerus, with 21 cases. There were only three cases of BM occurred in the extremities, two in the radius and one in the tibial diaphysis. This dissemination pattern reflects the red bone marrow vascularization, facilitating the implantation and growth of tumor cells. 14

Ideally, patients with BM should undergo a single procedure to enable early full weight-bearing and lasting the patient's life expectancy. 22 Patients with a life expectancy lower than 3 months can undergo non-surgical or less invasive treatments, while those with a longer life expectancy may benefit from more complex surgical procedures for tumor control and functional improvement. 23

In the current study, 88% of the patients (n = 124) underwent bone reconstruction with endoprosthesis. Postoperative complications occurred in 44 subjects, including 68% clinical and 10% surgical complications. A total of 4 patients presented prosthesis infections, with 2 requiring implant exchanges. Teixeira et al. 2 evaluated 64 patients undergoing surgery for BM. Of these, 17 (26.6%) presented complications, including 10 surgical, 4 clinical, and 3 combined complications. 2 Kumar et al. 24 reported 4 infections, 2 dislocations, and 2 aseptic loosening cases in 32 patients undergoing proximal femoral endoprosthesis procedures.

Approximately 60% of the patients with BM present comorbidities, especially cardiovascular, respiratory, and metabolic disorders. 5 Bindels et al. 25 associated early postoperative complications with factors such as fast-growing tumors, multiple BMs, pathological fractures, lower limb surgeries, hypoalbuminemia, hyponatremia, and leukocytosis. 25 Preoperative RT increases the risk of postoperative complications, predominantly when performed up to 2 months before surgery. 26 Although diabetes and RT were deemed risk factors, they had no association with complications.

Anemia negatively affects cancer patient survival regardless of the primary tumor. 9 10 However, in the present study we did not observe an association between hemoglobin levels and postoperative complications. Neither did leukocytosis from infections and inflammations show any correlation with complications. Nonetheless, there was an association between increased neutrophil counts and the risk of postoperative complications: for every 100 additional neutrophils, there was a 1% increase in the chance of complications.

The overall 1-year survival rate was of 34.7% (n = 49), with a median survival rate of 5 months. In patients with NLR ≥ 2 ( *p* < 0.001), survival decreased from 92.3 to 62.5% at 3 months, and from 61.5 to 31.3% after 1 year. Although the mechanisms of interaction between cancer and inflammatory factors require elucidation, studies 9 10 show that elevated NLR has an association with poor survival in several neoplasms, such as multiple myeloma and gastric, colorectal, lung, breast, and endometrial cancers. A meta-analysis of 40,559 patients with malignant tumors found that an elevated NLR, that is, higher than 4, results in worse overall survival.

Wang et al. 27 selected 497 patients with BM from different carcinomas, including 225 subjects who underwent surgical treatment, and evaluated the prognostic implications of NLR. These authors divided NLR into ≤ 3.0 and > 3.0, and they observed that a high NLR was associated with a worse prognosis, especially in surgical patients. 27 In an analysis of 1,012 patients with BM, Thio et al. 28 defined the NLR and PLR cutoff points as 4 and 408 respectively. Subjects with high NLR and PLR presented lower survival at 3 months; 84.0% of those with low NLR were alive compared with 61.3% of those with high NLR, and 75.8% of those with low PLR were alive compared with 55.6% of those with high PLR. 28

The prognostic value of PLR has been extensively studied in recent years. However, the mechanisms linking high PLR to poor prognosis in cancer patients remains unknown. A meta-analysis 10 of 12,754 patients from 20 studies on PLR in solid tumors showed that cutoff values ranged from 150 to 300 and revealed a significant association between high PLR and lower overall survival, especially in patients with metastatic disease. In the present study, patients with PLR ≥ 209 presented reduced survival, from 69 to 59.3% at 3 months and from 40.2 to 25.9% after 1 year.

The use of different cutoffs for NLR and PLR is controversial. Advanced disease stages, with higher inflammation levels, may result in increased NLR or PLR, indicating that the cutoff points may be higher. Furthermore, the prognostic values of NLR and PLR and their optimal cutoffs may vary among tumors; therefore, specific analyses are required.

The current study has limitations, including a statistical approach to define the optimal cutoff values for NLR and PLR based on data rather than hypotheses. The retrospective nature of the study did not enable the determination of uniform criteria for treatment or surgery, leading to the exclusion of patients not submitted to surgery, with BM in the axial skeleton, or with multiple myeloma. Thus, our conclusions apply to patients with BM in long bones and surgical candidates.

Certain factors, such as age, gender, ethnicity, chemotherapy, antibiotic therapy, transfusions, and infection may affect neutrophil, lymphocyte, or platelet counts. However, since the prognostic value of NLR and PLR is unclear, we believe that pretreatment counts reflect the patient's immune and inflammatory response without identifying the individual factors influencing them.

## Conclusion

In the current study, we did not observe a positive association regarding NLR, PLR and postoperative complication rates. However, we detected a strong correlation of increased NLR and PLR values with life expectancy reduction from the third month postoperatively.
