# Supplementary material for: Correlation of the Neutrophil-to-lymphocyte and Platelet-to-lymphocyte Ratios with Postoperative Complications and Survival in Surgery for Bone Metastasis of the Appendicular Skeleton
Source: Rev Bras Ortop (Sao Paulo). 2025 Apr 11;60(1):s00451804497. doi: 10.1055/s-0045-1804497 (PMC12020561; doi:10.1055/s-0045-1804497)
Supplement: Related texts

# Research article: Correlação das razões neutrófilo-linfócito e plaqueta-linfócito com complicações e sobrevida pós-operatórias na cirurgia para metástase óssea do esqueleto apendicular

**Keywords:** complicações pós-operatórias, linfócitos, metástase neoplásica, neutrófilos, plaquetas, procedimentos cirúrgicos operatórios

## Abstract

**Objetivo** Analisar, em metástases de ossos longos, a incidência de complicações e sobrevida pós-operatórias até um ano, e correlacioná-las com a razão neutrófilo-linfócito (RNL) e a razão plaqueta-linfócito (RPL).

**Métodos** Revisão de 160 prontuários de pacientes submetidos a cirurgias por metástases ósseas no esqueleto apendicular. Além das características epidemiológicas, foram determinados os valores da RNL e da RPL, que foram correlacionados com sobrevida e complicações.

**Resultados** Mulheres representaram 64,5% da amostra, com tumor primário na mama em 62,6%. O fêmur proximal foi o osso mais acometido. A sobrevida média foi de 13,2 meses, e a taxa de sobrevida em 1 ano, de 34,7%. Ressecção tumoral com endoprótese foi o procedimento mais comum. A taxa de complicação pós-operatória foi de 10%, e o tempo médio para a ocorrência de complicações pós-operatórias foi de 27,9 (variação: 0–140) dias. Foi identificada uma associação da variável neutrófilos com as complicações pós-operatórias ( *p* = 0,04): a cada 100 unidades a mais de neutrófilos, houve aumento de 1% nas chances de complicações pós-cirúrgicas. Os valores médios da RNL e da RPL foram, respectivamente, de 5,3 (variação: 0,2–30,7) e de 199,7 (variação: 32,1–676,7). Os pacientes com RNL ≥ 2 ( *p* < 0,001) apresentaram diminuição na sobrevida de 92,3 para 62,5% no terceiro mês, e de 61,5 para 31,3% em 1 ano. Aqueles com RPL ≥ 209 ( *p* < 0,001) apresentaram diminuição na sobrevida de 69 para 59,3% no terceiro mês, e de 40,2 para 25,9% em 1 ano.

**Conclusão** Não houve associação positiva entre RNL e RPL com complicações pós-operatórias, mas houve uma forte correlação da RNL e da RPL elevadas com a redução da expectativa de vida dos pacientes a partir do terceiro mês de pós-operatório.

## Introdução

O câncer é um problema de saúde pública, com cerca de 626 mil novos casos no Brasil em 2020. 1 A doença óssea metastática é a neoplasia maligna mais comum do esqueleto, e ocupa o terceiro lugar na disseminação de adenocarcinomas, após o pulmão e o fígado. 2 3 As metástases ósseas (MOs) ocorrem principalmente no esqueleto axial (80%), na pelve e no fêmur, e afetam especialmente mulheres acima da quarta década de vida. 4 5 A maioria das MOs originam-se da mama, da próstata ou do pulmão. 4 5

A presença de MO é a principal causa de morbidade em pacientes com câncer avançado, 6 e seu controle é essencial para melhorar a qualidade de vida, a dor e a independência do paciente. 2 O tratamento é multidisciplinar, e inclui medidas clínicas ou cirúrgicas.

A cirurgia busca estabilizar fraturas iminentes ou patológicas, ao promover a redução da dor, a melhora da função do membro, a deambulação precoce e ao evitar as complicações do decúbito prolongado. 2 A decisão cirúrgica deve considerar a localização, a extensão da metástase, a resposta a terapias adjuvantes, o quadro clínico e a expectativa de vida. 6 A taxa de complicações do tratamento da MO correlaciona-se à idade avançada, às comorbidades clínicas, à imunodeficiência, à desnutrição, à internação prolongada e à irradiação local. 7

A inflamação é um marco do câncer: em alguns tumores, precede a malignização; em outros, o tumor induz a resposta inflamatória, o que favorece o seu crescimento. 8 Biomarcadores inflamatórios, como a razão neutrófilo-linfócito (RNL, contagem de neutrófilos dividida pela de linfócitos) e a razão plaqueta-linfócito (RPL, contagem de plaquetas dividida pela de linfócitos), têm valor prognóstico em diversos tipos de câncer. 9 O aumento da RNL correlaciona-se a menor sobrevida em neoplasia gástrica, colorretal, pulmonar, de mama, de endométrio e em mieloma múltiplo. 10 Nesse contexto, a RNL e a RPL podem ser usadas para prever a sobrevida e as complicações pós-operatórias em pacientes com MO. 2

Este estudo visa identificar o perfil epidemiológico dos pacientes com metástase em ossos longos, a incidência de complicações, a taxa de sobrevida pós-operatória e a correlação da RNL e RPL com complicações e sobrevida em até um ano de pós-operatório.

## Materiais e Métodos

Trata-se de um estudo observacional retrospectivo, longitudinal, analítico e descritivo. Os pacientes foram selecionados a partir do livro de registro cirúrgico do Serviço de Oncologia Ortopédica do Hospital Erasto Gaertner, e os dados foram extraídos por meio de revisão dos prontuários eletrônicos Tasy (Philips Healthcare, Best, Holanda), em conformidade com a Lei Geral de Proteção de Dados Pessoais (LGPD, lei n° 13.709/2018). A coleta abrangeu pacientes operados no período de 1° de janeiro de 2010 a 31 de dezembro de 2019.

O perfil epidemiológico incluiu variáveis como idade, gênero, sítio primário do tumor, localização da MO, procedimento cirúrgico, presença de outras metástases, comorbidades e radioterapia (RT) prévia. Foram determinadas a RNL e a RPL e sua correlação com a sobrevida e as complicações pós-operatórias.

Os critérios de inclusão foram pacientes operados para o tratamento de MO do esqueleto apendicular, com confirmação diagnóstica de neoplasia maligna óssea metastática por exame histológico ou imunohistoquímico. E os critérios de exclusão foram prontuários incompletos, pacientes submetidos a cirurgia de revisão em outra instituição, e ausência de hemograma pré-operatório.

Os valores da RNL e RPL foram obtidos do hemograma pré-operatório, realizado no laboratório do hospital até 72h antes da cirurgia. Em pacientes submetidos a múltiplas cirurgias ortopédicas por MO, consideraram-se apenas os dados da primeira.

## Análise Estatística

Os dados foram organizados em planilha do programa Excel (Microsoft Corp., Redmond, WA, Estados Unidos) e analisados com o programa Stata/SE (StataCorp LLC, College Station, TX, Estados Unidos), versão 14.1. As variáveis quantitativas foram expressar por valores de média, desvio padrão, mediana, mínimo, máximo e intervalo interquartil (IIQ). As variáveis categóricas foram expressas por frequências absolutas e percentuais. Para analisar fatores os associados às complicações pós-operatórias, foram ajustados modelos de regressão logística. A significância de cada variável foi avaliada por meio do teste de Wald. A medida de associação estimada foi a razão de chances (RC) com intervalos de confiança de 95% (IC95%).

Os pontos de corte para a RNL e a RPL foram determinados pelo programa Cutoff Finder, 11 que identifica os pontos de corte que produzem diferenças significativas nos testes de *log-rank* . Esse processo assegura que os pontos de corte selecionados correspondam aos valores da RNL e da RPL que mais se relacionam com os desfechos clínicos selecionados. Valores de *p* < 0,05 indicaram significância estatística.

## Resultados

## Perfil Epidemiológico

Foram avaliados 160 prontuários entre 2010 e 2019. Destes, 16 foram excluídos por falta de dados ou perda de seguimento, 2, por ausência de confirmação de metástase no exame anatomopatológico, e 1, por cirurgia em outro serviço. Foram incluídas 154 cirurgias, sendo 12 em pacientes com múltiplas metástases. Para a análise dos dados, considerou-se apenas a primeira, totalizando 141 procedimentos.

A média de idade foi de 61,5 (variação: 25–89) anos, e a sobrevida média, de 13,2 (0–99,6) meses. Os parâmetros laboratoriais médios estavam dentro da normalidade, conforme os valores de referência para o hemograma normal. 12 A RNL foi de 5,3 (variação: 0,2–30,7) e a RPL, de 199,7 (variação: 32,1–676,7; Tabela 1 ).

**Tabela 1:** Variáveis epidemiológicas quantitativas

Variável	n	Média	Desvio padrão	Mediana	Mínimo	Máximo	Intervalo interquartil
Idade (anos)	141	61,5	12	62	25	89	54–69
Sobrevida (meses)	141	13,2	18,8	5	0	99,6	1,9–16,9
Neutrófilos	141	6.451	3.094	5.762	137	19.272	.4276–8.398
Linfócitos	141	1.542	779	1.315	301	4.518	999–1.958
Leucócitos	141	8.657	3.486	8.100	980	21.900	6.340–10.800
Plaquetas	141	25.2579	104.642	241.000	63.000	596.000	174.000–308.000
Razão neutrófilo-linfócito	141	5,3	4,5	4,2	0,2	30,7	2,7–6,3
Razão plaqueta-linfócito	141	199,7	118,8	171,6	32,1	676,7	114,9–248

Em relação ao gênero, 64,5% (n = 91) dos pacientes eram mulheres. O fêmur foi o osso mais acometido (n = 112), seguido do úmero (n = 21). Quanto ao procedimento cirúrgico, 67% dos pacientes foram submetidos à ressecção do tumor com substituição por endoprótese de quadril. A maioria (80,1%) tinha múltiplas MOs, e 51,1% apresentavam metástases viscerais. Os demais dados estão resumidos na Tabela 2 .

**Tabela 2:** Variáveis epidemiológicas categóricas

Variável	Classificação	n	%
Gênero	Feminino	91	64,50%
	Masculino	50	35,50%
Anatomia	Fêmur diafisário	3	2,10%
	Fêmur distal	10	7,10%
	Fêmur proximal	99	70,20%
	Rádio	2	1,40%
	Tíbia diafisária	1	0,70%
	Tíbia proximal	5	3,50%
	Úmero diafisário	8	5,70%
	Úmero distal	2	1,40%
	Úmero proximal	11	7,80%
Operação	Endoprótese de quadril	95	67,40%
	Endoprótese de joelho	15	10,60%
	Endoprótese de ombro	10	7,10%
	Fixação de fratura	6	4,30%
	Ressecção artroplástica	4	2,80%
	Endoprótese diafisária: fêmur	3	2,10%
	Endoprótese de cotovelo	2	1,40%
	Amputação	1	0,70%
	Desarticulação	1	0,70%
	Endo fêmur total	1	0,70%
	Endoprótese diafisária: úmero	1	0,70%
	Ressecção do rádio proximal	1	0,70%
	Ressecção intralesional	1	0,70%
Presença de outras metástases viscerais	Não	72	51,10%
	Sim	69	48,90%
Presença de outras metástases ósseas	Não	28	19,90%
	Sim	113	80,10%
Diabete	Não	110	78,00%
	Sim	31	22,00%
Radioterapia prévia	Não	136	96,50%
	Sim	5	3,50%

A distribuição e a categorização do tumor primário estão na Tabela 3 . No gênero feminino, o sítio primário mais comum foi mama, com 62,6% (n = 57), e no masculino, o tumor primário mais comum foi o de próstata, com 38% (n = 19). Em ambos, a segunda neoplasia mais comum foi a de pulmão.

**Tabela 3:** Gênero e diagnóstico primário

Diagnóstico primário	Gênero				Geral	
	Feminino		Masculino			
	n	%	n	%	n	%
Câncer de mama	57	62,60%			57	40,40%
Câncer de próstata			19	38,00%	19	13,50%
Câncer de pulmão e anexos	9	9,8	9	18,00%	18	12,70%
Câncer de rim	5	5,50%	9	18,00%	14	9,90%
Diagnóstico primário desconhecido	7	7,70%	1	2,00%	8	5,70%
Câncer de cólon	1	1,10%	2	4,00%	3	2,10%
Câncer de endométrio	3	3,30%			3	2,10%
Câncer gástrico	2	2,20%	1	2,00%	3	2,10%
Câncer de bexiga	2	2,20%			2	1,40%
Câncer de esôfago			2	4,00%	2	1,40%
Câncer de pele			2	4,00%	2	1,40%
Câncer de faringe			1	2,00%	1	0,70%
Câncer de gastrointestinal	1	1,10%			1	0,70%
Hemangiopericitoma intracraneano	1	1,10%			1	0,70%
Câncer hepático			1	2,00%	1	0,70%
Câncer de laringe			1	2,00%	1	0,70%
Melanoma	1	1,10%			1	0,70%
Câncer de orofaringe			1	2,00%	1	0,70%
Câncer de pâncreas	1	1,10%			1	0,70%
Câncer de tireoide			1	2,00%	1	0,70%
Câncer de útero e anexos	1	1,10%			1	0,70%
Total	91	100%	50	100%	141	100%

## Complicações Pós-operatórias

Conforme descrito na Tabela 4 , o tempo médio para a ocorrência de complicações pós-operatórias foi de 27,9 (variação: 0–140) dias. A taxa de complicações foi de 31,2% (n = 44). A Tabela 5 mostra que as complicações mais comuns foram pneumonia (n = 10) e infecção da ferida operatória (n = 5). Ao todo, 4 pacientes apresentaram infecção profunda, sendo 2 casos associados à luxação da prótese. Todos foram submetidos à limpeza e desbridamento cirúrgico, e 2 pacientes foram submetidos a uma nova cirurgia para a troca do implante.

**Tabela 4:** Tempo até a ocorrência da complicação pós-operatória

Variável	n	Média	Desvio padrão	Mediana	Mínimo	Máximo	Intervalo interquartil
Tempo até a complicação pós-operatória (dias)	44	27,9	31,7	15	0	140	3–42

**Tabela 5:** Incidência de complicações pós-operatórias

Variável	Classificação	n	%
Complicações pós-operatórias	Não	97	68,8%
	Sim	44	31,2%
Tipo de complicação pós-operatória (n = 44)	Pneumonia	10	22,7%
	Infecção de ferida operatória	5	11,3%
	Hemorragia do trato gastrointestional	4	9,1%
	Infecção de prótese	4	9,1%
	Luxação de prótese	4	9,1%
	Oclusão arterial aguda de membro inferior	2	4,5%
	Sangramento de ferida operatória	2	4,5%
	Tromboembolismo pulmonar	2	4,5%
	Choque hemodinâmico	2	4,5%
	Recidiva local	2	4,5%
	Hipercalcemia	2	4,5%
	Deiscência com exposição de material	1	2,3%
	Infecção do trato urinário	1	2,3%
	Síndrome da resposta inflamatória sistêmica	1	2,3%
	Trombose venosa profunda	1	2,3%

## Avaliação da Associação entre os Fatores e a Ocorrência de Complicações Pós-operatórias

Para cada variável, testou-se a hipótese nula de associação com a probabilidade de complicação pós-operatória contra a hipótese alternativa de que havia associação. A Tabela 6 apresenta os fatores demográficos e clínicos e as complicações pós-operatórias.

**Tabela 6:** Fatores demográficos e clínicos e complicações pós-operatórias

Variável	Classificação	Total	Complicação pós-operatória		p*	RC (IC95%)
			Não	Sim		
Idade (anos)	Média ± DP	141	60,7 ± 12,5	63,3 ± 10,9	0,238	1,02 (0,99–1,05)
Gênero	Masculino	50	39 (78,0%)	11 (22,0%)		
	Feminino	91	58 (63,7%)	33 (36,3%)	0,083	2,02 (0,91–4,46)
Presença de outras metástases viscerais	Não	72	46 (63,9%)	26 (36,1%)		
	Sim	69	51 (73,9%)	18 (26,1%)	0,201	1,60 (0,78–3,29)
Presença de outras metástases ósseas	Não	28	19 (67,9%)	9 (32,1%)		
	Sim	113	78 (69%)	35 (31,0%)	0,905	1,06 (0,44–2,57)
Diabete	Não	110	76 (69,1%)	34 (30,9%)		
	Sim	31	21 (67,7%)	10 (32,3%)	0,886	0,94 (0,40–2,21)
Radioterapia prévia	Não	136	94 (69,1%)	42 (30,9%)		
	Sim	5	3 (60,0%)	2 (40,0%)	0,668	0,67 (0,11–4,16)
Neutrófilos (a cada 100)	Mediana (IIQ)	141	5.550 (3.760–7.900)	6.941 (4.933–8.689)	0,040	1,01 (1,001–1,02)
Linfócitos (a cada 100)	Mediana (IIQ)	141	1.280 (974–1.940)	1.361 (1.058–2.005)	0,925	1,00 (0,96–1,05)
Leucócitos (a cada 100)	Média ± DP	141	8.280 ± 3.246	9.487 ± 3.876	0,062	1,01 (1,00–1,02)
Plaquetas (a cada 10.000)	Mediana (IIQ)	141	237.000 (181.000–304.000)	243.500 (154.000–310.000)	0,797	1,00 (0,96–1,03)
Hemoglobina	Média ± DP	141	11,9 ± 2,0	11,3 ± 1,8	0,145	0,87 (0,72–1,05)
**Razão neutrófilo- linfócito**	**Mediana (IIQ)**	**141**	**4,1 (2,5–5,8)**	**4,5 (3,2–6,9)**	**0,534**	**1,03 (0,95–1,11)**
**Razão plaqueta-linfócito**	**Mediana (IIQ)**	**141**	**167 (116–269)**	**177 (105–242)**	**0,534**	**0,999 (0,996–1,002)**

**Abreviaturas:** DP, desvio-padrão; IC95%, intervalo de confiança de 95%; IIQ, intervalo interquartil; RC, razão de chances.

**Nota:** *Modelo de regressão logística e teste de Wald; *p* < 0,05.

Não houve correlação da hemoglobina, da diabete ou da RT prévia com as complicações. A presença de outras metástases, viscerais ou ósseas, também não teve correlação com as complicações. Com relação aos neutrófilos tomados isoladamente, houve associação significativa com a probabilidade de complicação ( *p* = 0,040): a cada 100 unidades a mais, a chance de complicação aumenta em 1%. A RNL e RPL não foram consideradas fatores de risco para complicação.

## Óbito

## Análise Descritiva do Tempo de Sobrevida

A Fig. 1 apresenta a curva de Kaplan-Meier do tempo de sobrevida dos pacientes e a mediana, que foi de 5 meses, com taxa de sobrevida de 34,7% (n = 49) em um ano.

**Fig. 1:** Curva de Kaplan-Meier com o tempo de sobrevida dos pacientes (intervalo de confiança de 95%, IC95%). Definição de pontos de corte para a razão neutrófilo-linfócito (RNL) e a razão plaqueta-linfócito (RPL) associados ao tempo de sobrevida.

Os valores de corte mais significativos foram RNL= 2 e RPL = 209. A Tabela 7 e a Fig. 2 apresentam as taxas de sobrevida e as curvas de Kaplan-Meier considerando o ponto de corte para RNL = 2. Testou-se a hipótese nula de que as curvas de sobrevida eram iguais para RNL < 2 e RNL ≥ 2 contra a hipótese alternativa de que eram diferentes. A partir do terceiro mês de pós-operatório, a sobrevida dos pacientes com RNL ≥ 2 ( *p* < 0.001) diminui de 92,3 para 62,5%, e aos 12 meses, de 61,5 para 31,3% ( Tabela 8 ).

**Tabela 7:** Taxa de sobrevida considerando o ponto de corte de 2 para a razão neutrófilo-linfócito

Variável	Classificação	Total	Óbito				*p* *
			Não		Sim		
			n	%	n	%	
Razão neutrófilo- linfócito	< 2	13	2	15,4%	11	84,6%	< 0,001
	≥ 2	128	0	0%	128	100%	

**Nota:** *Teste de *log-rank* ; *p* < 0,05.

**Fig. 2:** Curva de sobrevida considerando o ponto de corte de 2 para a RNL.

**Tabela 8:** Percentuais de sobrevida estimados pela curva de Kaplan-Meier para RNL < 2 e ≥ 2

Tempo	% de sobrevida	
	RNL <2	RNL ≥2
0 = operação	100%	100%
1 mês	92,3%	92,2%
3 meses	92,3%	62,5%
6 meses	69,2%	41,4%
12 meses	61,5%	31,3%

Abreviatura: RNL, razão neutrófilo-linfócito.

Para a RPL, testou-se a hipótese nula de que as curvas de sobrevida eram iguais para casos com RPL < 209 e RPL ≥ 209 contra a hipótese alternativa de que eram diferentes. Semelhante à RNL, pacientes com RPL ≥ 209 ( *p* < 0.001) apresentaram menor sobrevida, com a taxa diminuindo de 69 para 59,3% no terceiro mês, e de 40,2 para 25,9% após 1 ano de pós-operatório ( Tabela 9 ). A Tabela 10 e a Fig. 3 mostram os resultados e as curvas de sobrevida de Kaplan-Meier considerando o ponto de corte para RPL = 209.

**Tabela 9:** Taxa de sobrevida considerando o ponto de corte de 209 para a razão plaqueta-linfócito

Variável	Classificação	Total	Óbito				*p* *
			Não		Sim		
			n	%	n	%	
Razão plaqueta-linfócito	< 209	87	2	2,3%	85	97,7%	0,019
	≥ 209	54	0	0%	54	100%	

**Nota:** *Teste de *log-rank* ; *p* < 0,05.

**Tabela 10:** Percentuais de sobrevida estimados pela curva de Kaplan-Meier para RPL < 209 e ≥ 209

Tempo	% de sobrevida	
	RPL < 209	RPL ≥ 209
0 = operação	100%	100%
1 mês	81,6%	79,6%
3 meses	69,0%	59,3%
6 meses	49,4%	35,2%
12 meses	40,2%	25,9%

Abreviatura: RPL, razão plaqueta-linfócito.

**Fig. 3:** Curva de sobrevida considerando o ponto de corte de 209 para a RPL.

## Discussão

Anualmente, mais de 18 milhões de casos de câncer são registrados no mundo, e cerca de 50% desenvolverão doença metastática avançada. 13 14 Com o avanço do tratamento oncológico, a sobrevida dos pacientes aumenta e, paralelamente, o número de MOs também. Estima-se que em 2030 a prevalência de MO nos Estados Unidos alcance 600 mil indivíduos. 4

Este estudo incluiu 141 pacientes submetidos a tratamento cirúrgico para MO, com média de idade de 61,5 (variação: 25–89) anos e predominância de mulheres (64,5%; n = 91). Meohas et al. 15 observaram maior incidência em mulheres acima dos 40 anos, assim como Li et al. 16

Hernández et al. 17 analisaram 382.733 pacientes com tumores sólidos primários. A incidência de MO foi de 6,9% (n = 26.250), com média de idade de 64 anos. Os tumores mais prevalentes e que tiveram maior suscetibilidade para MO foram os cânceres de mama, de próstata e de pulmão. 17 Em outro estudo, 18 com 85.296 pacientes com MO, a prevalência foi de 64,2% para os tumores de mama, de 59,89% para os de próstata, de 52,85% para os da nasofaringe, de 35,82% para os de pulmão e de 35,10% para o câncer renal. Semelhante à literatura, nesta pesquisa, 66% das MOs se originaram dos tumores de mama, próstata e pulmão.

Mais de 80% das MOs localizam-se no esqueleto axial, e acometem principalmente as vértebras, as costelas e os quadris. 19 No esqueleto apendicular, localizam-se frequentemente nas regiões proximais dos membros, ao passo que lesões abaixo do joelho e do cotovelo são raras e associadas a neoplasias de pulmão, rim e tireoide. 20 Em um estudo 21 com 171 pacientes, 58 apresentaram lesões apendiculares: 73,8% no fêmur proximal, 58,3% no úmero proximal, e somente 4 casos na tíbia. Nesta análise, o osso mais acometido foi o fêmur, com 112 casos, seguido do úmero, com 21 casos. Houve somente 3 MOs de extremidades: duas no rádio e uma na diáfise tibial. Esse padrão de disseminação reflete a vascularização da medula óssea vermelha, que facilita a implantação e o crescimento de células tumorais. 14

Idealmente, pacientes com MO somente devem ser submetidos a um procedimento que permita suporte total de peso precoce e dure a expectativa de vida prevista. 22 Pacientes com expectativa de vida inferior a 3 meses podem ser tratados de forma não cirúrgica ou com técnicas menos invasivas, ao passo que aqueles com expectativa maior podem se beneficiar de procedimentos cirúrgicos mais complexos para controle tumoral e melhora da função. 23

Neste estudo, 88% dos pacientes (n = 124) passaram por reconstrução óssea com endoprótese. Complicações pós-operatórias ocorreram em 44 pacientes; 68% de natureza clínica e 10%, cirúrgica. Ao todo, 4 pacientes apresentaram infecção de prótese, com 2 trocas de implante. Teixeira et al. 2 avaliaram 64 pacientes operados por MOs. Destes, 17 (26,6%) apresentaram complicações: 10 complicações cirúrgicas, 4 clínicas e 3 combinadas. 2 Kumar et al., 24 relataram 4 infecções, 2 luxações e 2 solturas assépticas em 32 pacientes submetidos a procedimentos de endoprótese de fêmur proximal.

Cerca de 60% dos pacientes com MO apresentam comorbidades, especialmente cardiovasculares, respiratórias e metabólicas. 5 Bindels et al. 25 associaram complicações pós-operatórias precoces a fatores como tumores de crescimento rápido, múltiplas MOs, fratura patológica, cirurgia em membros inferiores, hipoalbuminemia, hiponatremia e leucocitose. 25 A RT pré-operatória aumenta o risco de complicações pós-operatórias, especialmente quando realizada até 2 meses antes da cirurgia. 26 Diabetes e RT foram avaliados como fatores de risco, mas sem associação com complicações.

A anemia afeta negativamente a sobrevida de pacientes com câncer, independentemente do tumor primário. 9 10 Contudo, neste estudo, não houve associação entre níveis de hemoglobina e complicações pós-operatórias. A leucocitose, associada a infecções e inflamações, também não mostrou correlação com complicações. Entretanto, identificou-se uma associação entre o aumento dos neutrófilos e o risco de complicação pós-operatória: a cada 100 unidades a mais de neutrófilos, houve um aumento de 1% na chance de complicação.

A taxa de sobrevida global em 1 ano foi de 34,7% (n = 49), com mediana de 5 meses. Nos pacientes com RNL ≥ 2 ( *p* < 0.001), a sobrevida diminuiu de 92,3 para 62,5% em 3 meses, e de 61,5 para 31,3% em 1 ano. Embora os mecanismos de interação entre o câncer e os fatores inflamatórios necessitem de esclarecimento, estudos 9 10 mostram que a RNL elevada está associada a menor sobrevida em vários tipos de câncer, como gástrico, colorretal, pulmonar, mamário, endometrial e mieloma múltiplo. Uma metanálise com 40.559 pacientes com tumores malignos descobriu que RNL elevada, acima de 4, está associada a pior sobrevida global.

Wang et al. 27 selecionaram 497 pacientes com MO de diferentes carcinomas, 225dos quais foram tratados cirurgicamente, e avaliaram as implicações prognósticas da RNL. Eles dividiram a RNL em ≤ 3,0 e > 3,0, e observaram que RNL alta estava associada a pior prognóstico, especialmente nos pacientes cirúrgicos. 27 Em uma análise de 1.012 pacientes com MO, Thio et al. 28 definiram pontos de corte de 4 para a RNL e de 408 para a RPL. Pacientes com RNL e RPL elevadas apresentaram menor sobrevida aos 3 meses: 84,0% dos com RNL baixa estavam vivos, comparados a 61,3% dos com RNL alta; e 75,8% dos com RPL baixa, comparados a 55,6% dos com RPL alta. 28

O valor prognóstico da RPL tem sido amplamente estudado nos últimos anos, mas os mecanismos que ligam RPL elevada a mau prognóstico em pacientes com câncer ainda são desconhecidos. Uma metanálise 10 com 12.754 pacientes de 20 estudos sobre RPL em tumores sólidos mostrou que os valores de corte variaram de 150 a 300, e revelou uma associação significativa entre RPL elevada e menor sobrevida global, especialmente em pacientes com doença metastática. No presente estudo, pacientes com RPL ≥ 209 apresentaram redução na sobrevida: de 69 para 59,3% no terceiro mês, e e de 40,2 para 25,9% após 1 ano.

O uso de diferentes pontos de corte para a RNL e A RPL é controverso. O estágio avançado da doença, com maior nível de inflamação, pode resultar em uma RNL ou uma RPL mais elevadas, o que indica que os pontos de corte podem ser maiores. Além disso, os valores prognósticos da RNL e da RPL e seus pontes de corte ideais podem variar entre diferentes tumores, o que indica que análises específicas são necessárias.

Este estudo tem limitações, como o uso de uma abordagem estatística para definir os valores de corte ideais para RNL e RPL, baseada em dados, não em hipóteses. Devido à natureza retrospectiva, não houve critérios uniformes para o tratamento ou a cirurgia, e pacientes não submetidos a cirurgia, com MO no esqueleto axial ou com mieloma múltiplo foram excluídos. Assim, nossas conclusões se aplicam a pacientes com MO em ossos longos e candidatos à cirurgia.

Fatores como idade, gênero, raça, quimioterapia, antibióticos, transfusões e infecções podem afetar as contagens de neutrófilos, linfócitos ou plaquetas. No entanto, como os valores prognóstico da RNL e da RPL não estão elucidados, acreditamos que as contagens pré-tratamento refletem a resposta imunológica e inflamatória dos pacientes, sem identificar os fatores individuais que as influenciaram.

## Conclusão

Neste estudo, não foi verificada associação positiva da RNL e da RPL com taxas de complicações pós-operatórias. Contudo, observou-se forte correlação do aumento da RNL e da RPL com a redução da expectativa de vida dos pacientes a partir do terceiro mês de pós-operatório.
